# *Eupatorium lindleyanum* DC. Suppresses Cytokine Storm by Inhibiting NF-κB and PI3K–Akt Signaling in Sepsis-Associated and Virus-Related Acute Lung Injury

**DOI:** 10.3390/cimb48030333

**Published:** 2026-03-21

**Authors:** Chen Luo, Peilin He, Yan Yang, Lian Xia, Wenjie Xu, Daike Zou, Yiduo Feng, Lian Duan, Junjie Deng, Yong Jing, Xianqin Luo

**Affiliations:** 1 College of Chinese Materia Medica, Chongqing University of Chinese Medicine, Chongqing 402760, China; 2 College of Traditional Chinese Medicine, Chongqing Medical University, Chongqing 400016, China; 3 Institute of Pharmacology and Toxicology, Chongqing Academy of Chinese Materia Medica, Chongqing 400065, China

**Keywords:** *Eupatorium lindleyanum* DC., hyperoside, sepsis-induced acute lung injury, COVID-19, cytokine storm

## Abstract

Cytokine storm is a central pathogenic mechanism underlying sepsis-induced acute lung injury (SALI) and severe coronavirus disease 2019 (COVID-19), yet effective therapeutic strategies remain limited. *Eupatorium lindleyanum* DC. (EL), a traditional Chinese medicinal herb, has been reported to possess anti-inflammatory, antioxidant, and antiviral-related activities; however, its protective mechanisms in SALI and virus-associated inflammatory lung injury remain incompletely understood. In this study, an integrated strategy combining computational prediction and experimental validation was employed to investigate the therapeutic potential and underlying mechanisms of EL. The chemical constituents of EL were characterized by UPLC–Q–TOF/MS, followed by network pharmacology, molecular docking, and molecular dynamics analyses to predict key targets and signaling pathways. A cecal ligation and puncture (CLP)-induced SALI rat model was used to evaluate lung histopathology, pulmonary edema, cytokine production, and inflammatory signaling activation. In parallel, LPS-stimulated RAW264.7 macrophages were used to assess cytokine secretion and pathway regulation in vitro. In addition, a SARS-CoV-2 pseudovirus-induced mouse model was employed to further evaluate the in vivo relevance of the representative bioactive compound hyperoside in pseudovirus-associated lung injury. A total of 32 active compounds and 697 putative targets were identified, among which 116 were associated with sepsis and COVID-19. In vivo, EL markedly alleviated lung injury, reduced the lung coefficient and wet/dry ratio, and suppressed excessive production of proinflammatory cytokines and activation of key signaling proteins. In vitro, EL dose-dependently inhibited TNF-α and IL-6 secretion and regulated the PI3K–Akt and NF-κB signaling pathways. Notably, hyperoside showed favorable predicted interactions with PI3K–Akt pathway-related targets (EGFR, PI3K, and Akt), while molecular dynamics simulations supported stable interactions with several COVID-19-related targets, including ACE2, Mpro, and RdRp. Furthermore, hyperoside significantly alleviated SARS-CoV-2 pseudovirus-associated lung injury, reduced ACE2 protein expression, and downregulated EGFR, PI3K, and Akt mRNA levels in vivo. Collectively, these findings indicate that EL exerts protective effects through multi-component, multi-target, and multi-pathway mechanisms, and support its potential value for further investigation in SALI and virus-associated inflammatory lung injury.

## 1. Introduction

Sepsis represents a severe clinical condition in which infection causes a disrupted host immune response, leading to failure of multiple organs [1]. During this progression, the lung becomes one of the most vulnerable organs [2], with acute lung injury (ALI) occurring in more than 40% of patients [3]. The severity of sepsis is largely attributed to the activation and amplification of the inflammatory cascade, namely the cytokine storm syndrome (CSS), which is marked by the excessive production and release of proinflammatory cytokines [4]. Although the pathogenesis of COVID-19-induced organ damage remains incompletely understood, evidence such as hemophagocytosis, elevated systemic cytokines, and clinical responses to immunosuppressants in critically ill patients indicates that excessive inflammation is a key pathogenic factor [5]. In particular, cytokine storm syndrome (CSS) has been identified as a core pathological factor responsible for the exacerbation of COVID-19 and even death in patients, as revealed by numerous clinical and basic research studies [6,7,8]. Therefore, sepsis-induced acute lung injury (SALI) and COVID-19-related lung injury share highly similar pathogenic mechanisms, particularly in cytokine storm-mediated immune-inflammatory responses. This mechanistic overlap provides a theoretical basis for exploring common therapeutic strategies.

*Eupatorium lindleyanum* DC. (EL), a traditional Chinese medicinal herb, is renowned for its pharmacological effects, including anti-inflammatory, antibacterial, antiviral, lipid-lowering, protective effects against ALI, and antitussive activities [9,10]. Its therapeutic spectrum coincides with the pathological features of both SALI and COVID-19. Supporting evidence includes reports that EL syrup was confirmed by the Chinese Center for Disease Control and Prevention to prevent coronavirus infection [11], EL compound capsules showed efficacy against respiratory infectious diseases [12], EL extracts inhibited tobacco mosaic virus infection [13], and EL ethanol extracts exhibited anti-inflammatory effects by modulating the TLR4/NF-κB/NLRP3 pathway and reshaping gut microbiota to provide multi-modal protection against ALI [14]. These findings collectively suggest that EL may exert both anti-inflammatory and antiviral effects, targeting the shared core mechanism of SALI and COVID-19-cytokine storm. However, systematic investigations into its precise protective effects and molecular mechanisms in SALI remain lacking.

Based on these insights, we hypothesized that EL may alleviate SALI by suppressing inflammatory signaling pathways, thereby reducing proinflammatory cytokine release and attenuating cytokine storm-mediated injury. To test this hypothesis, we integrated network pharmacology, molecular docking, and molecular dynamics simulations, combined with in vivo validation using a SALI rat model and in vitro studies in LPS-stimulated macrophages.

## 2. Materials and Methods

### 2.1. Materials and Reagents

*Eupatorium lindleyanum* DC. was sourced from Xuyi Defeng Traditional Chinese Medicine Planting Co., Ltd. (Huai’an, China) and its identity was verified by Professor Xianyuan He from the College of Traditional Chinese Medicine, Chongqing Medical University. The species name and taxonomic information were further confirmed in World Flora Online (ID: wfo-0000021725). According to our previously reported method, the EL materials were extracted twice by reflux with 70% ethanol under reduced pressure and heating, after which the concentrated solution was freeze-dried to obtain the total EL extract [10] (Supplementary Figure S1).

Dexamethasone acetate tablets (Batch No. 200923) were obtained from Zhejiang Xianju Pharmaceutical Co., Ltd. (Hangzhou, China). Fetal bovine serum was obtained from GeZhe Biotechnology (Batch No. 3022A, Umedium, Hefei, China). ELISA kits for TNF-α (EHJ-20039r), IL-6 (EHJ-20746r), and IL-1β (EHJ-20537r) were sourced from Xiamen Huijia Biotechnology Co., Ltd. (Xiamen, China). Kits for GM-CSF (E-EL-R0008), IFN-γ (E-EL-R0009), IL-5 (E-EL-R0558), and IL-18 (E-EL-R0567) were supplied by Elabscience Biotechnology Co., Ltd. (Wuhan, China). Antibodies targeting Akt (4691), IκBα (4812), and p-IκBα (2859) were obtained from Cell Signaling Technology (Boston, MA, USA). Antibodies against P65 (TA5006), p-P65 (TP70621), PI3K (T40115), and p-PI3K (TA4372) were purchased from Abmart (Shanghai, China). In addition, EGFR (YM8344), p-EGFR (YM8664), p-Akt (YP0006), and GAPDH (YN5585) were supplied by ImmunoWay (San Jose, CA, USA) [10,15].

### 2.2. UPLC-Q-TOF/MS Analysis

An ultra-fast liquid chromatography device, 30AT (Shimadzu Corporation, Shimadzu, Japan), and TripleTOFTM 5600 LC/MS (AB SCIEX, Framingham, MA, USA) were used to determine the components of EL. For chromatographic analysis, a Kinetex C18 column (Φ 2.1 mm × 100 mm; 2.6 μm) was used at 30 °C. The mobile phase was a mixture of acetonitrile (A) and water (B), both containing 0.1% formic acid, using gradient elution (1 min: 90% A; 7 min: 15% A; 11 min: 15% A; 11.5 min: 90% A; 15.1 min: stop); the flow rate was 0.3 mL/min. Combined with mass spectrometry, we used an EFI ion source under two modes, ESI_ Positive and ESI_ Negative, and the dynamic background subtraction to trigger the information association acquisition mode. Then we got the UPLC-Q-TOF/MS data and analyzed it using Analyst1.6 software and Peakview 1.2TM.

### 2.3. Network Pharmacology Investigation of the Active Constituents of EL

The 2D chemical structures of the detected compounds identified by UPLC-Q-TOF/MS were retrieved from the PubChem database. Potential targets were predicted using the SEA Search Server and SwissTargetPrediction, and only targets with a prediction probability greater than zero were retained. All predicted targets were standardized using the UniProt database to ensure consistent gene and protein nomenclature. Disease-associated targets related to acute lung injury (ALI) were collected from the GeneCards, DrugBank, and OMIM databases and the Therapeutic Target Database (TTD). For the GeneCards database, only genes with a relevance score greater than 1 were retained to improve the specificity of disease-related targets. All retrieved targets were merged, duplicates were removed, and gene names were standardized using UniProt. The overlapping targets between EL-related targets and disease-associated targets were identified and visualized using Venn diagram analysis, and the intersection genes were considered potential therapeutic targets. These intersecting targets were imported into the STRING database to construct a protein–protein interaction (PPI) network with a confidence score greater than 0.9. The resulting network was visualized and analyzed using Cytoscape 3.7.2, and hub genes were identified using the CytoHubba plugin based on the Degree algorithm. Functional enrichment analysis was performed using the clusterProfiler package in R 4.3.3. KEGG pathway enrichment analysis was conducted using the enrichKEGG function, and Benjamini–Hochberg correction was applied to control the false discovery rate. Pathways with adjusted *p*-values < 0.05 were considered significantly enriched. The significantly enriched pathways were ranked according to their adjusted *p*-values, and 15 inflammation-related signaling pathways were selected for further analysis and visualization based on their known roles in inflammatory responses and cytokine regulation.

### 2.4. Animal Treatment

Male Sprague–Dawley rats (6–8 weeks, 180–220 g) from the Animal Center of Chongqing Medical University (License No. SYXK [Yu] 2022-0010) were acclimatized for one week and randomly assigned into six groups (*n* = 6): sham surgery (Sham), the CLP-induced ALI model (MG), dexamethasone (DEX, 5 mg/kg), and low/medium/high-dose EL (6/12/18 g/kg). All animals received oral administration of the designated dose once daily for seven consecutive days. Two hours after the final administration, cecal ligation and puncture (CLP) was performed to induce sepsis-associated acute lung injury (SALI), as previously described [16].

All surgical procedures were conducted under sodium pentobarbital anesthesia with a strict aseptic technique. Twenty-four hours after CLP, rats were euthanized, and serum and lung tissues were collected. Serum and lung samples were stored at −80 °C, while portions of lung tissues were fixed in 4% paraformaldehyde for histopathological examination. All procedures adhered to institutional guidelines for animal care and were approved by the Animal Ethics Committee of Chongqing Medical University.

### 2.5. Cell Culture

RAW264.7 cells (Wuhan Pricella Biotechnology Co., Ltd. Wuhan, China) were cultured in DMEM medium supplemented with 10% heat-inactivated fetal bovine serum (FBS) and 1% penicillin–streptomycin, and maintained at 37 °C in a humidified atmosphere containing 5% CO_2_. Cells were subcultured up to passage 5 before use in experiments. For cytotoxicity analysis, the RAW264.7 cells were exposed to graded concentrations of EL for 24 h. Cell viability was assessed using the CCK-8 assay: 10 µL of CCK-8 solution was added to each well, followed by incubation for 1 h at 37 °C under 5% CO_2_. Absorbance at 450 nm was then measured with a microplate reader, and the results were normalized to the untreated control group (set as 100% viability). To establish the inflammatory model, the RAW264.7 cells were stimulated with LPS for 24 h. To evaluate the protective effect of EL, the cells were allowed to adhere overnight, pretreated with EL for 2 h, and then exposed to LPS for an additional 24 h.

### 2.6. Evaluation of the Pharmacodynamic Properties of EL

Body weight was measured 24 h after the final dosing. Following bronchoalveolar lavage, the lungs were excised and weighed to determine the lung coefficient (lung weight/body weight × 100%). The wet weight (W) of the left upper lobe was recorded, after which the tissue was dried at 60 °C for 48 h to obtain the dry weight (D), and the W/D ratio was calculated. The left lower lobe was fixed in 4% paraformaldehyde, dehydrated through graded ethanol, cleared with xylene, and embedded in paraffin. Sections of 5 µm thickness were prepared, stained with hematoxylin and eosin (H&E), and examined under a light microscope. Lung tissue across the entire section was assessed for inflammatory injury and scored according to the following criteria: normal (0), hemorrhage (0–1), peribronchial cell infiltration (0–1), interstitial edema (0–2), pneumocyte hyperplasia (0–3), and intra-alveolar infiltration (0–3) [17].

### 2.7. Enzyme-Linked Immunosorbent Assay (ELISA)

After the treatments, serum and lung tissues were harvested. Concentrations of IL-6, IL-1β, and TNF-α were quantified using ELISA kits from Xiamen Huijia Biotechnology Co., Ltd. (Xiamen, China) following the manufacturer’s protocols. Levels of GM-CSF, IFN-γ, IL-5, and IL-18 were measured with ELISA kits supplied by Wuhan AmyJet Scientific Inc. (Wuhan, China).

### 2.8. Western Blot Analysis

Consistently with earlier findings, Western blot analysis was conducted [18]. In brief, proteins were extracted from cells or lung tissues using RIPA lysis buffer supplemented with protease and phosphatase inhibitors (Beyotime Biotechnology Co., Ltd., Shanghai, China). Protein concentrations were determined by a BCA assay, and samples were resolved by SDS-PAGE. Following electrophoretic transfer onto PVDF membranes (Millipore, Burlington, MA, USA), the blots were blocked with 5% skim milk for 1 h, incubated with primary antibodies overnight at 4 °C, and then exposed to HRP-linked secondary antibodies. Protein bands were visualized using ECL detection reagents (Advansta, San Jose, CA, USA). Antibodies included RTK (1:5000), p-RTK (1:5000), PI3K (1:1000), p-PI3K (1:1000), Akt (1:1000), p-Akt (1:1000), TLR4 (1:1000), IκBα (1:1000), p-IκBα (1:1000), P65 (1:1000), p-P65 (1:1000) and GAPDH (1:5000). Quantification used ImageJ 1.54f.

### 2.9. Molecular Docking

Molecular docking simulations were performed to explore the potential interactions between the identified bioactive compounds and key target proteins. Docking calculations were conducted using AutoDock Vina 1.5.6 integrated in the AMDock platform. The three-dimensional structures of target proteins were obtained from the Protein Data Bank (PDB). Prior to docking, protein structures were prepared by removing water molecules and other non-essential heteroatoms, followed by the addition of polar hydrogen atoms and assignment of Gasteiger charges. Ligand structures were obtained from the PubChem database and subjected to energy minimization before docking. The grid box was centered on the coordinates of the co-crystallized ligand and sized to fully cover the binding pocket (approximately 20 × 20 × 20 Å). The exhaustiveness parameter of AutoDock Vina was set to 8 to ensure sufficient conformational sampling during docking. Binding affinity scores were calculated using the AutoDock Vina scoring function. To visualize and analyze the docking conformations and interactions between ligands and target proteins, PyMOL 3.1.6.1 was employed for structural visualization and figure preparation. To verify the reliability of the docking protocol, a re-docking validation was performed using the co-crystallized ligand of the target protein. The ligand was extracted from the original crystal structure and re-docked into the same binding pocket under identical docking parameters. The root-mean-square deviation (RMSD) between the experimental and redocked ligand conformations was calculated using PyMOL. The obtained RMSD value was 0.306 Å, indicating excellent reproducibility and reliability of the docking protocol.

### 2.10. Molecular Dynamics Simulation

Molecular dynamics (MD) simulations were performed using the GROMACS software package 2025.3. The protein–ligand complex obtained from molecular docking was placed in a cubic simulation box with a minimum distance of 1.0 nm between the protein surface and the box boundary. The system was solvated using the SPC water model, and Na^+^ and Cl^−^ ions were added to neutralize the system. The CHARMM36 force field was employed for the MD simulations. Energy minimization was first performed using the steepest descent algorithm, followed by the conjugate gradient method to eliminate unfavorable steric contacts and stabilize the system. After energy minimization, the system was equilibrated in two phases. First, NVT equilibration was carried out to stabilize the temperature of the system. Subsequently, NPT equilibration was performed to stabilize pressure and system density under periodic boundary conditions. Following equilibration, production molecular dynamics simulations were carried out for 100 ns. The structural stability and dynamic behavior of the protein–ligand complexes were evaluated using root mean square deviation (RMSD), root mean square fluctuation (RMSF), the radius of gyration (Rg), solvent-accessible surface area (SASA), and hydrogen bond analysis. Trajectory processing and structural analyses were conducted using the built-in tools of GROMACS.

### 2.11. Data and Statistical Analysis

Data are presented as the mean ± SD and were analyzed using GraphPad Prism 9. Comparisons between two groups were performed using an unpaired *t*-test, whereas multiple groups were evaluated by one-way ANOVA. A *p*-value < 0.05 was considered statistically significant. Significance markers were defined as ^#^ *p* < 0.05 and ^##^ *p* < 0.01 versus the Sham or CG group and * *p* < 0.05 and ** *p* < 0.01 versus the MG group.

## 3. Results

### 3.1. EL Treatment Attenuated the Inflammatory Response in Rats with CLP-Induced ALI

**Figure 1:**
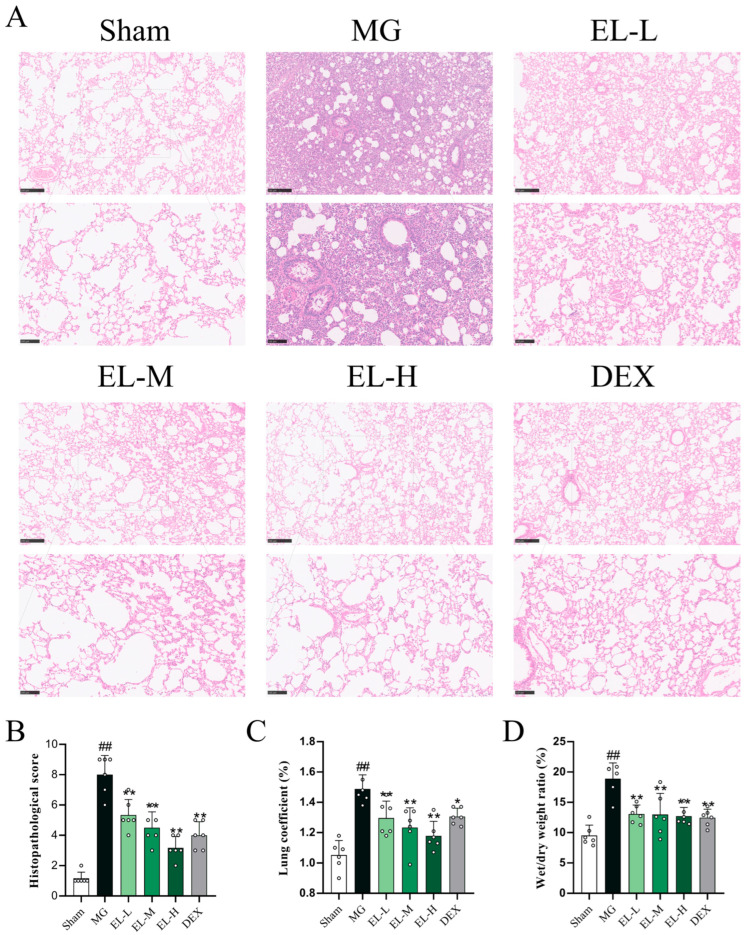
EL decreased the inflammation in SALI rats. (**A**) Representative photomicrographs of HE staining of lung tissues in each group (100×, 200× magnification). (**B**) Lung injury scoring scale (*n* = 6). (**C**) Lung coefficient. (**D**) Lung wet/dry weight ratio (*n* = 6). ^##^ *p* < 0.01 compared to Sham. * *p* < 0.05 and ** *p* < 0.01 compared to MG.

H&E staining showed that EL administration substantially mitigated CLP-induced pulmonary edema, hemorrhage, inflammatory cell infiltration, and thickening of the alveolar septa, along with a notable decrease in overall lung injury scores (*p* < 0.01, Figure 1A,B). Consistently, EL dose-dependently decreased the lung coefficient and the wet/dry weight ratio (*p* < 0.01, Figure 1C,D).

### 3.2. EL Treatment Inhibited the NF-κB Signaling Pathway and Attenuated the Cytokine Storm in Rats with CLP-Induced ALI

**Figure 2:**
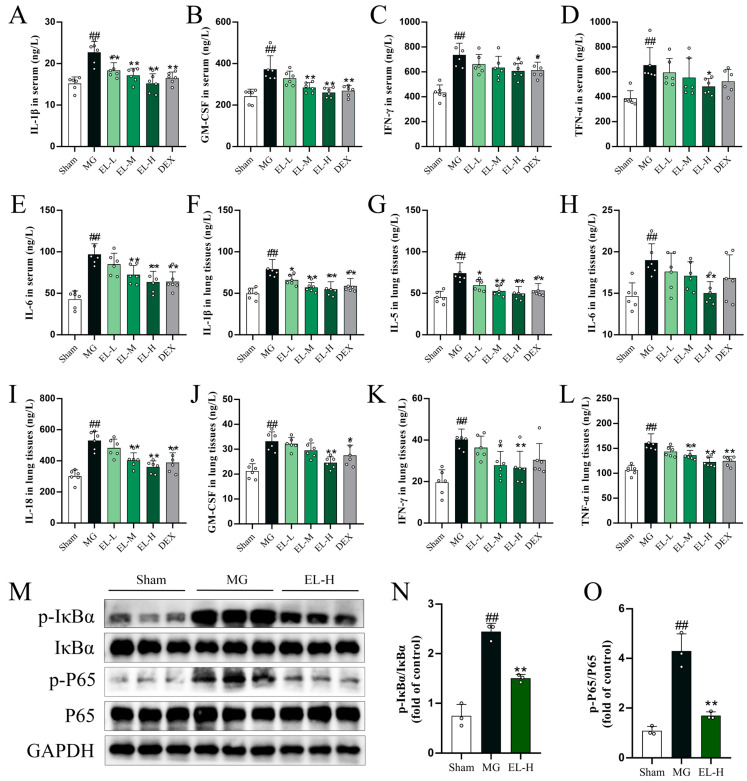
EL inhibits NF-κB activation and attenuates the cytokine storm in SALI rats. (**A**) Serum IL-1β levels (*n* = 6). (**B**) Serum GM-CSF levels (*n* = 6). (**C**) Serum IFN-γ levels (*n* = 6). (**D**) Serum TNF-α levels (*n* = 6). (**E**) Serum IL-6 levels (*n* = 6). (**F**) Lung tissue IL-1β levels (*n* = 6). (**G**) Lung tissue IL-5 levels (*n* = 6). (**H**) Lung tissue IL-6 levels (*n* = 6). (**I**) Lung tissue IL-18 levels (*n* = 6). (**J**) Lung tissue GM-CSF levels (*n* = 6). (**K**) Lung tissue IFN-γ levels (*n* = 6). (**L**) Lung tissue TNF-α levels (*n* = 6). (**M**) Western blot analysis of NF-κB pathway proteins (*n* = 3). (**N**) Relative densitometric ratio of p-IκBα/IκBα (*n* = 3). (**O**) Relative densitometric ratio of p-P65/P65 (*n* = 3). ^##^ *p* < 0.01 compared to Sham. * *p* < 0.05 and ** *p* < 0.01 compared to MG.

A CLP-induced rat model of sepsis was constructed to assess the pharmacological effects of EL. Cytokine levels in serum and lung tissues were quantified using ELISA, and Western blotting was applied to examine the expression of NF-κB pathway-related proteins, including IκBα and P65, in lung tissues. CLP induction led to a pronounced elevation of IL-1β, GM-CSF, IFN-γ, TNF-α, and IL-6 levels in serum (*p* < 0.01, Figure 2A–E), as well as IL-1β, IL-5, IL-6, IL-18, GM-CSF, IFN-γ, and TNF-α in lung tissues (*p* < 0.01, Figure 2F–L), and elevated the protein expression of p-IκBα and p-P65 in lung tissues (*p* < 0.05, Figure 2M–O). Treatment with EL or DEX significantly reduced these levels (*p* < 0.05), with EL at a high dose (EL-H) showing superior efficacy to DEX in cytokine suppression. Among these, TNF-α, IL-1β, and IL-6 were the most prominent cytokines during the cytokine storm. Collectively, these findings indicate that both EL and DEX effectively inhibited activation of the NF-κB signaling pathway, attenuated the CLP-induced cytokine storm in vivo, and thereby mitigated the associated inflammatory cascade.

### 3.3. EL Mitigated the LPS-Triggered Inflammatory Response in Macrophages by Reducing Cytokine Secretion

**Figure 3:**
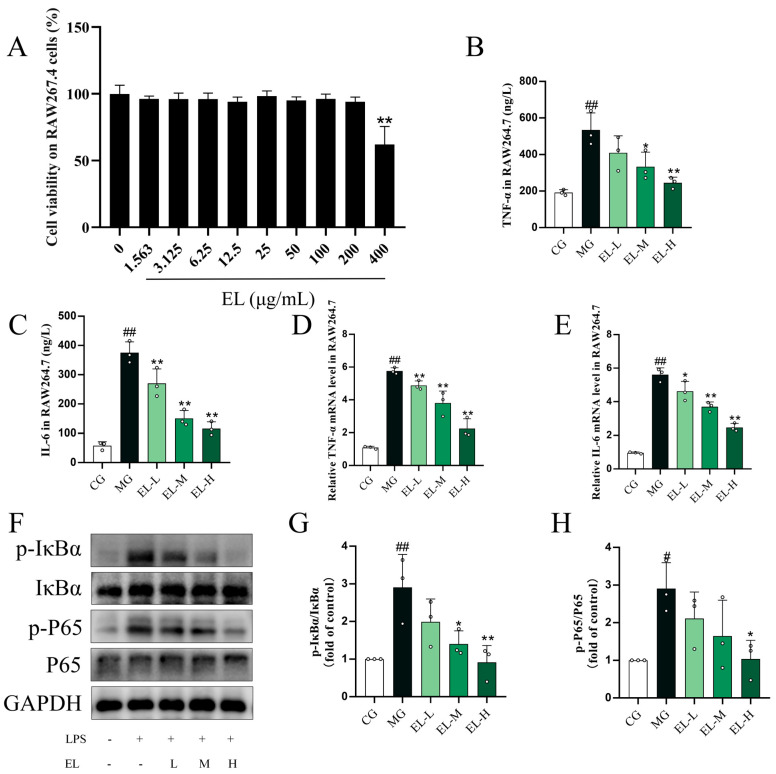
EL attenuates LPS-induced inflammatory responses in RAW264.7 cells (*n* = 3). (**A**) CCK-8 assay showing the effects of EL (0–400 μg/mL) on RAW264.7 cell viability. (**B**) TNF-α in LPS-stimulated RAW264.7 cells. (**C**) IL-6 in LPS-stimulated RAW264.7 cells. (**D**) TNF-α mRNA expression levels. (**E**) IL-6 mRNA expression levels. (**F**) Representative Western blot images of p-IκBα, IκBα, p-P65, and P65. (**G**) Quantitative analysis of p-IκBα/IκBα expression. (**H**) Quantitative analysis of p-P65/P65 expression. ^#^ *p* < 0.05 and ^##^ *p* < 0.01 compared to CG. * *p* < 0.05 and ** *p* < 0.01 compared to MG.

CCK-8 assay confirmed that EL exhibited no cytotoxicity in RAW264.7 cells at concentrations ranging from 0.1563 to 200 μg/mL, providing a rationale for selecting anti-inflammatory doses (Figure 3A). In LPS-stimulated RAW264.7 cells, EL (50, 100, and 200 μg/mL) significantly suppressed both the secretion (*p* < 0.01, Figure 3B,C) and transcription (*p* < 0.01, Figure 3D,E) of TNF-α and IL-6. Furthermore, EL attenuated the LPS-induced upregulation of IκBα and P65, two key nodes of the NF-κB signaling pathway, in RAW264.7 cells (*p* < 0.05, Figure 3F–H).

### 3.4. Predicted Therapeutic Mechanisms of EL Against Sepsis and COVID-19

**Figure 4:**
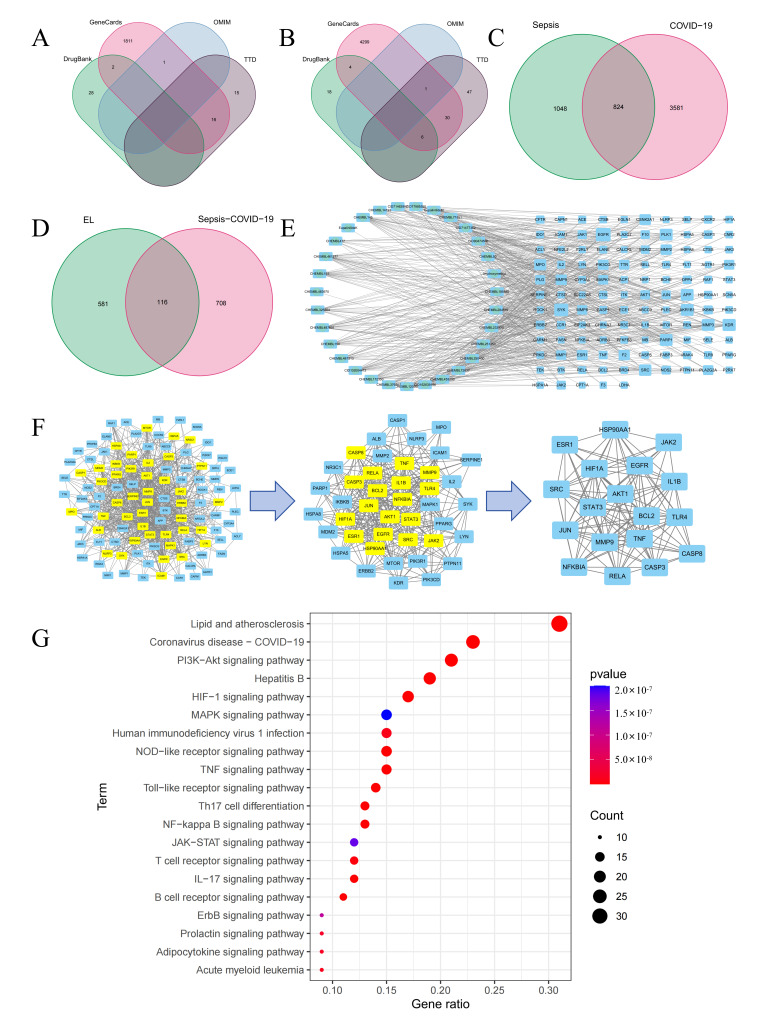
The potential target pathways related to the against sepsis and COVID-19 activity of EL. (**A**) Sepsis-related targets included in various platforms. (**B**) COVID-19-related targets included in various platforms. (**C**) The intersection of targets between sepsis and COVID-19. (**D**) Overlap of EL potential targets and sepsis–COVID-19-related targets. (**E**) Network diagram of EL components and related targets. (**F**) Identify the key PPI network using a scoring-based evaluation approach. (**G**) Top 20 enriched KEGG pathways.

A total of 1873 putative targets associated with sepsis and 4405 putative targets associated with COVID-19 were identified (Figure 4A,B), among which 824 targets were shared (Figure 4C). EL was found to intersect with 116 of these common targets (Figure 4D). A protein–protein interaction (PPI) network highlighted 18 hub genes, including AKT1, TNF, TLR4, and JAK2 (Figure 4E,F). KEGG enrichment analysis further identified the PI3K-Akt signaling pathway as a key pathway underlying the therapeutic effect of EL in ALI (Figure 4G).

### 3.5. Molecular Docking of Hyperoside with Principal Targets Involved in the PI3K–Akt Pathway

**Figure 5:**
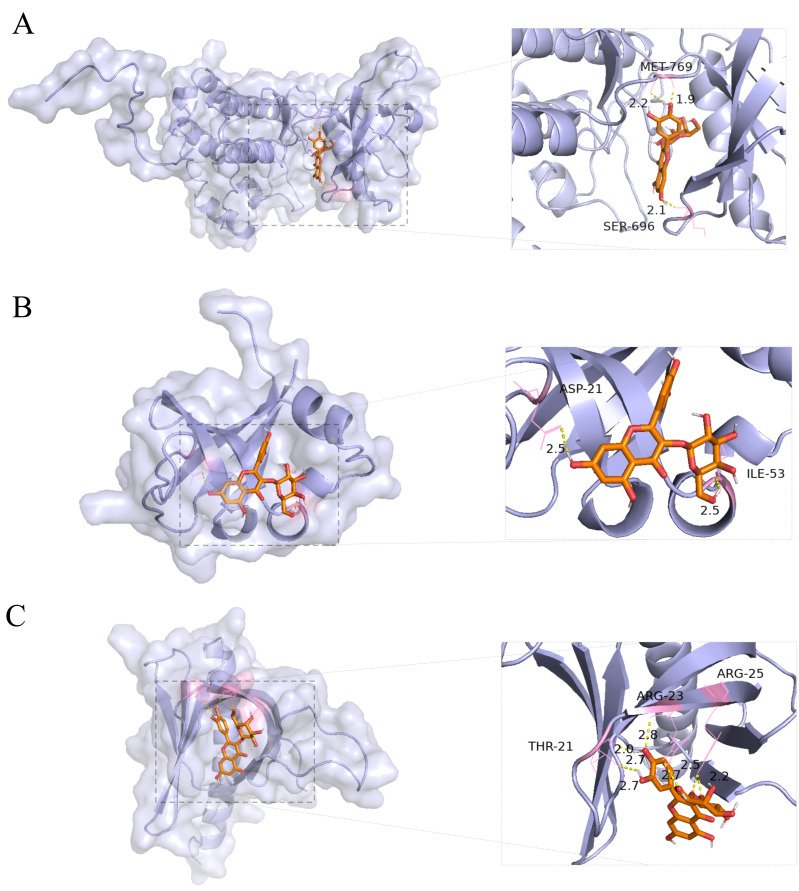
Molecular docking analysis. (**A**) Hyperoside with EGFR. (**B**) Hyperoside with PI3K. (**C**) Hyperoside with Akt.

The PPI network revealed that hyperoside was the only component capable of simultaneously targeting the key nodes of the PI3K-Akt signaling pathway, namely EGFR, PI3K, and Akt. To further evaluate these interactions, molecular docking analysis was performed. The results showed binding energies of −8.4, −7.0, and −6.7 kcal/mol with EGFR, PI3K, and Akt, respectively, suggesting favorable binding interactions between hyperoside and these targets (Figure 5A–C).

### 3.6. Molecular Dynamics Simulations of Hyperoside Bound to Key Targets of the PI3K-Akt Signaling Pathway

**Figure 6:**
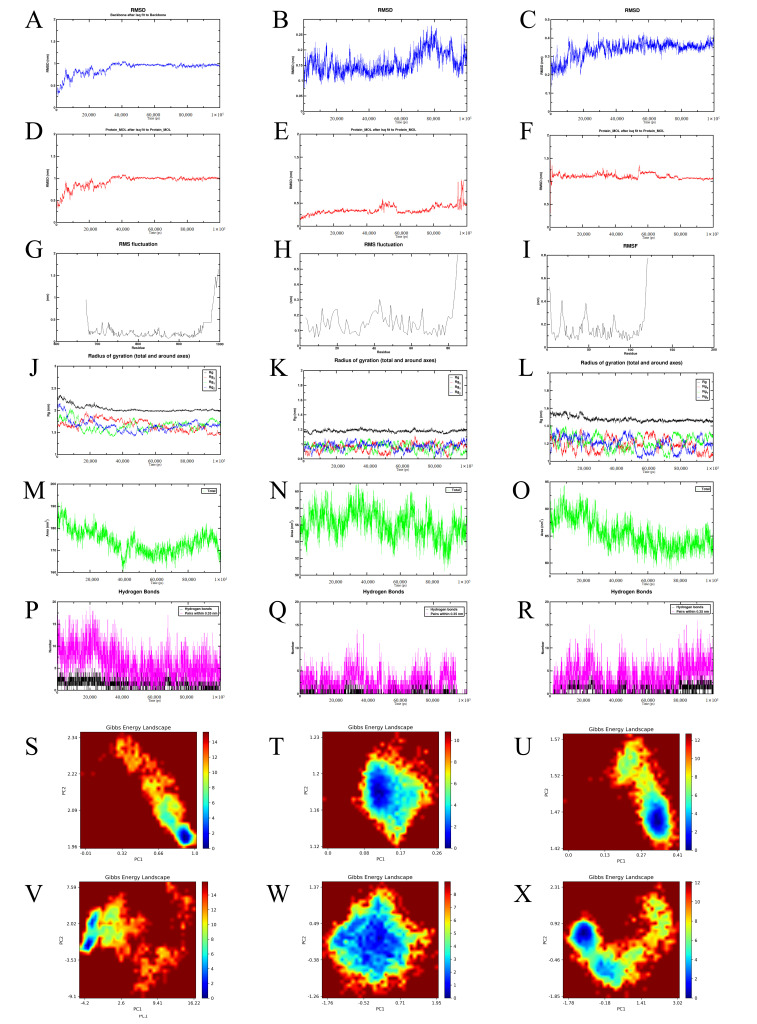
Molecular dynamics simulations of hyperoside bound to EGFR, PI3K, and Akt. RMSD trajectories showing structural stability of hyperoside-EGFR (**A**), hyperoside-PI3K (**B**), and hyperoside-Akt (**C**) complexes. RMSD convergence plots for hyperoside-EGFR (**D**), hyperoside-PI3K (**E**), and hyperoside-Akt (**F**). RMSF analysis illustrating residue flexibility in hyperoside-EGFR (**G**), hyperoside-PI3K (**H**), and hyperoside-Akt (**I**). Rg reflecting overall compactness of hyperoside-EGFR (**J**), hyperoside-PI3K (**K**), and hyperoside-Akt (**L**). Solvent-accessible surface area changes for hyperoside-EGFR (**M**), hyperoside-PI3K (**N**), and hyperoside-Akt (**O**) complexes. Hydrogen bond dynamics during simulation of hyperoside-EGFR (**P**), hyperoside-PI3K (**Q**), and hyperoside-Akt (**R**). Gibbs free energy landscapes of hyperoside-EGFR (**S**), hyperoside-PI3K (**T**), and hyperoside-Akt (**U**) complexes, showing dominant low-energy states. Magnified FEL maps of hyperoside-EGFR (**V**), hyperoside-PI3K (**W**), and hyperoside-Akt (**X**), depicting conformational distribution and stability.

In the molecular dynamics simulations, the root mean square deviation (RMSD) was used to assess the structural stability of the complexes throughout the simulation process. The hyperoside-EGFR and hyperoside-Akt complexes exhibited relatively stable conformations, whereas the hyperoside-PI3K complex showed notable conformational changes (Figure 6A–F). The root mean square fluctuation (RMSF), which reflects protein flexibility during the simulations, remained low for all three complexes except at the terminal regions, indicating good rigidity of the protein core structures (Figure 6G–I). The radius of gyration (Rg), representing the overall compactness of the proteins, remained stable across all three complexes, suggesting that no significant unfolding occurred during the simulations (Figure 6J–L). The solvent-accessible surface area (SASA), which reflects the solvent-exposed surface of the proteins, remained relatively stable, with a slight decrease observed in the hyperoside-EGFR complex (Figure 6M–O). Analysis of hydrogen bonds revealed that all three complexes maintained multiple hydrogen bonds over time, supporting stable interactions, although the hyperoside-PI3K complex exhibited greater fluctuations, indicating relatively dynamic binding (Figure 6P–R). Finally, the Gibbs free energy landscape, derived from principal component analysis (PCA), demonstrated that all three complexes adopted stable conformational states (Figure 6S–X).

### 3.7. Effects of EL on the PI3K-Akt Signaling Pathway

**Figure 7:**
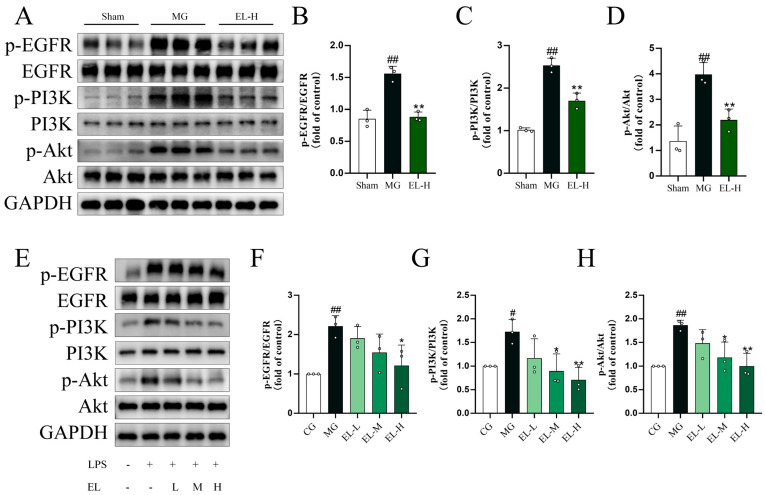
EL attenuates SALI in rats and LPS-induced inflammatory response in RAW264.7 macrophages by suppressing the PI3K-Akt signaling pathway (*n* = 3). (**A**) Western blot analysis of p-EGFR, EGFR, p-PI3K, PI3K, p-Akt, and Akt in lung tissues of CLP-induced ALI rats. Semi-quantitative analysis showing that CLP markedly increased the ratios of p-EGFR/EGFR (**B**), p-PI3K/PI3K (**C**), and p-Akt/Akt (**D**), while EL-H significantly reduced these levels. (**E**) Western blot detection of p-EGFR, EGFR, p-PI3K, PI3K, p-Akt, and Akt in RAW264.7 cells following LPS stimulation. Quantification showing that EL suppressed LPS-induced increases in p-EGFR/EGFR (**F**), p-PI3K/PI3K (**G**), and p-Akt/Akt (**H**) in a dose-dependent manner. ^#^ *p* < 0.05 and ^##^ *p* < 0.01 compared to CG. * *p* < 0.05 and ** *p* < 0.01 compared to MG.

Mechanistic investigations in the CLP-induced ALI rat model and in LPS-treated RAW264.7 macrophages showed that EL inhibited the PI3K–Akt signaling pathway in a dose-dependent manner (Figure 7A,E). In ALI rats, EL counteracted CLP-driven upregulation of EGFR (*p* < 0.01, Figure 7B), PI3K (*p* < 0.01, Figure 7C), and Akt (*p* < 0.01, Figure 7D). In RAW 264.7 cells, SLEL suppressed LPS-induced phosphorylation of EGFR (*p* < 0.05, Figure 7F), PI3K (*p* < 0.01, Figure 7G), and Akt (*p* < 0.01, Figure 7H).

### 3.8. Molecular Docking of Hyperoside with Three Key Targets of COVID-19 (ACE2, Mpro, and RdRp)

**Figure 8:**
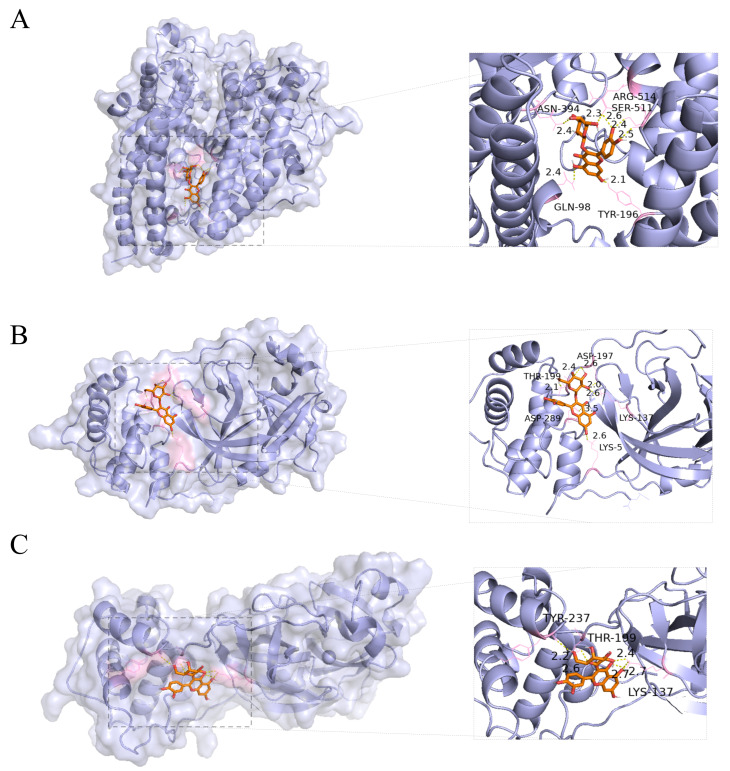
Molecular docking analysis. (**A**) Hyperoside with ACE2. (**B**) Hyperoside with Mpro. (**C**) Hyperoside with RdRp.

To further explore the potential antiviral relevance of hyperoside, molecular docking analysis was performed to evaluate its interactions with the key COVID-19-related targets ACE2, Mpro, and RdRp. The results showed predicted binding energies of −8.4, −7.7, and −9.4 kcal/mol with ACE2, Mpro, and RdRp, respectively (Figure 8A–C), suggesting favorable binding interactions between hyperoside and these targets.

### 3.9. Molecular Dynamics Simulations of Hyperoside with Two Key COVID-19 Targets (ACE2 and Mpro)

**Figure 9:**
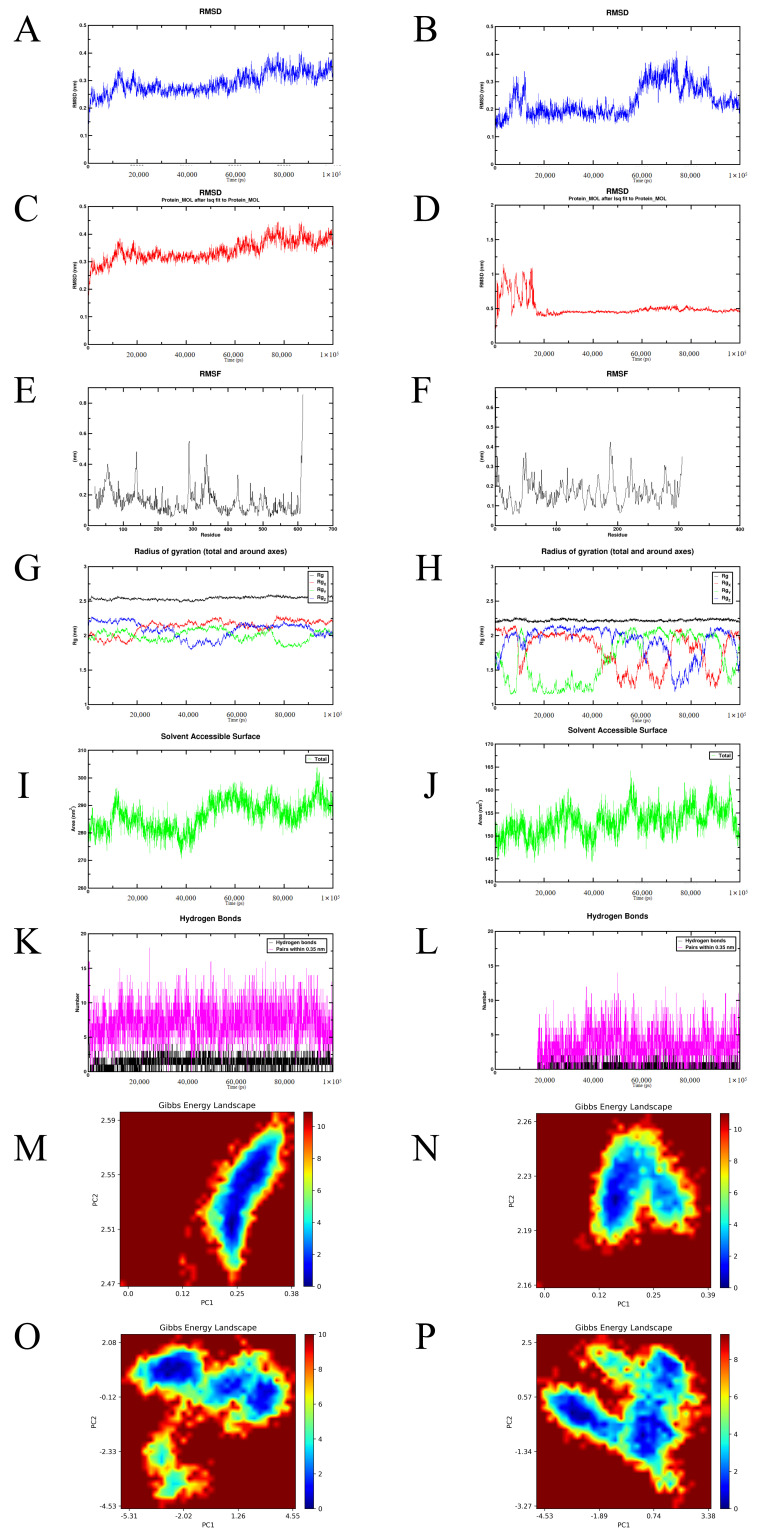
Molecular dynamics simulations of hyperoside with ACE2 and Mpro. RMSD trajectories showing structural stability of hyperoside-ACE2 (**A**) and hyperoside–Mpro (**B**) complexes. RMSD convergence plots for hyperoside-ACE2 (**C**) and hyperoside–Mpro (**D**). RMSF analysis illustrating residue flexibility for ACE2 (**E**) and Mpro (**F**). Radius of gyration (Rg) indicating conformational compactness of ACE2 (**G**) and Mpro (**H**) complexes. SASA changes in ACE2 (**I**) and Mpro (**J**) complexes. Hydrogen bond dynamics during simulations of hyperoside-ACE2 (**K**) and hyperoside–Mpro (**L**). Gibbs free energy landscapes of hyperoside-ACE2 (**M**) and hyperoside-Mpro (**N**) complexes, showing dominant low-energy states. Magnified FEL maps of hyperoside-ACE2 (**O**), and hyperoside-Mpro (**P**) depicting conformational distribution and stability.

To further assess the stability of hyperoside binding to the two major COVID-19 targets, ACE2 and Mpro, molecular dynamics simulations were conducted. The results demonstrated that hyperoside formed stable ligand–receptor conformations with both targets by occupying specific binding pockets. During the simulations, the hyperoside-ACE2 and hyperoside-Mpro complexes remained stable and compact (Figure 9A–D). The RMSF values of both complexes showed fluctuations mainly in a few loop regions, while the core structures remained relatively rigid (Figure 9E,F). The Rg was essentially stable, indicating good overall protein compactness after binding (Figure 9G,H). The solvent-accessible surface area (SASA) exhibited slight fluctuations for the hyperoside-ACE2 complex and even smaller variations for hyperoside-Mpro, suggesting a stable conformation (Figure 9I,J). Although the number of hydrogen bonds in both complexes fluctuated throughout the simulations, the hyperoside-Mpro complex showed smaller variations, with hydrogen bonds being more persistent and stable (Figure 9K,L). The Gibbs free energy landscapes derived from principal component analysis (Figure 9M–P) revealed that the ACE2 complex predominantly occupied a single, narrow energy basin, while the Mpro complex explored multiple low-energy conformations, suggesting conformational adaptability within a stable energetic framework. Collectively, these results indicate that hyperoside binds to both ACE2 and Mpro with structural stability but exhibits a stronger and more stable interaction with Mpro, potentially enhancing its inhibitory efficacy.

### 3.10. Hyperoside Mitigates SARS-CoV-2 Pseudovirus-Associated Lung Injury and Suppresses ACE2/EGFR–PI3K–Akt Signaling

**Figure 10:**
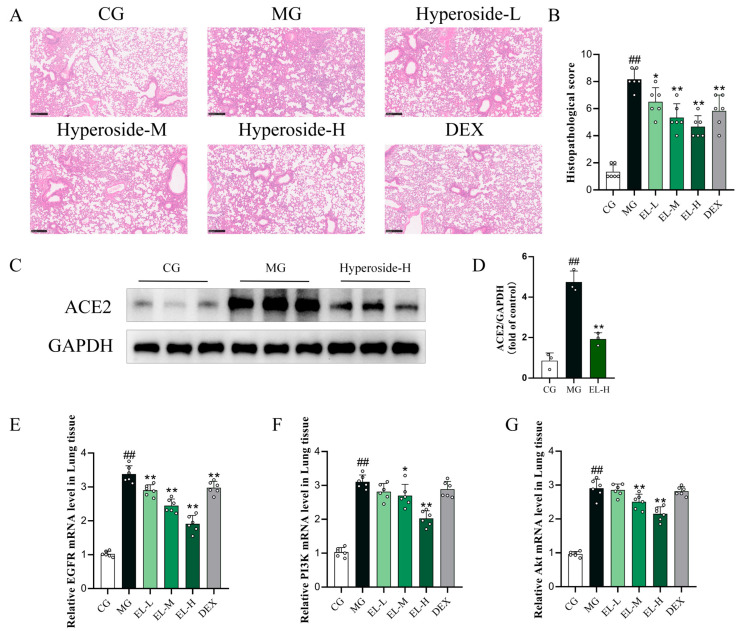
Hyperoside mitigates and suppresses SARS-CoV-2 pseudovirus-associated lung injury. ACE2/EGFR–PI3K–Akt signaling in vivo. (**A**) Representative H&E-stained lung sections from each group (CG, MG, hyperoside-L, hyperoside-M, hyperoside-H, and DEX) at 100× magnification. (**B**) Quantitative histopathological injury scores of lung tissues (*n* = 6). (**C**) Representative Western blot images showing ACE2 protein expression in lung tissues, with GAPDH as a loading control. (**D**) Densitometric analysis of ACE2 protein levels normalized to GAPDH. (**E**–**G**) Relative mRNA expression levels of EGFR (**E**), PI3K (**F**), and Akt (**G**) in lung tissues, as determined by RT-qPCR. Data are presented as mean ± SD. ^##^ *p* < 0.01 vs. CG; * *p* < 0.05 and ** *p* < 0.01 vs. MG.

To further evaluate the in vivo relevance of hyperoside in SARS-CoV-2 pseudovirus-associated lung injury, a pseudovirus-induced lung injury model was established in C57BL/6 mice. Histopathological examination revealed that pseudovirus challenge caused marked lung injury, characterized by alveolar structural disruption, inflammatory cell infiltration, and interstitial edema, compared with the control group (Figure 10A). Hyperoside treatment significantly alleviated these pathological changes in a dose-dependent manner, with the high-dose group (hyperoside-H) showing a protective effect comparable to that of DEX. Consistently, quantitative histological scoring showed that lung injury scores were significantly increased in the model group and markedly reduced following hyperoside administration (Figure 10B). Western blot analysis further showed that ACE2 protein expression was significantly upregulated in lung tissues after pseudovirus exposure, whereas high-dose hyperoside treatment markedly reduced ACE2 expression (Figure 10C,D). At the transcriptional level, pseudovirus challenge significantly increased the mRNA expression of EGFR, PI3K, and Akt in lung tissues. Hyperoside treatment reduced the expression of these genes, particularly in the medium- and high-dose groups (Figure 10E–G). These findings suggest that hyperoside alleviates SARS-CoV-2 pseudovirus-associated lung injury, potentially in association with reduced ACE2 expression and downregulation of EGFR/PI3K/Akt-related transcripts.

## 4. Discussion

SALI remains a major clinical challenge, with no effective pharmacological interventions currently available, particularly in the context of viral infections such as COVID-19. A common pathological hallmark of both conditions is the cytokine storm, characterized by excessive release of proinflammatory cytokines, among which TNF-α, IL-6, and IL-1β are particularly prominent. In this study, EL, a traditional Chinese medicinal herb, demonstrated significant protective effects against SALI through its multi-component and multi-target pharmacological actions.

Upon activation by pathogen- or damage-associated molecular patterns (PAMPs/DAMPs), cells release a range of cytokines, such as IL-6, IL-1β, TNF-α, IL-18, and IFN-γ, which collectively amplify the cytokine storm and contribute to extensive tissue damage [19,20]. Our results demonstrated that EL consistently lowered these cytokine levels in both in vivo and in vitro settings, Importantly, EL not only reduced systemic cytokine production but also attenuated macrophage hyperactivation, which represents the initial cellular trigger of cytokine storm. EL may reduce cytokine release by modulating the EGFR–PI3K–Akt–NF-κB inflammatory axis, thereby potentially decreasing macrophage activation. In the CLP-induced sepsis rat model, EL markedly attenuated pulmonary edema, thickening of the alveolar septa, hemorrhagic changes, and inflammatory cell infiltration, accompanied by reductions in the lung wet/dry ratio and overall injury scores. In LPS-stimulated RAW264.7 macrophages, EL significantly suppressed the release of TNF-α and IL-6, thereby attenuating macrophage-driven inflammatory responses. Given that macrophage hyperactivation plays a central role in the initiation of ALI and cytokine storm [21,22], and that elevated IL-6 and TNF-α levels strongly correlate with patient mortality [23], these results highlight the potential clinical relevance of EL in the management of SALI.

To further contextualize our findings, it is worth noting that SALI and severe COVID-19 share a highly similar inflammatory landscape dominated by macrophage-driven hyperinflammation and excessive cytokine production. This mechanistic convergence suggests a potential rationale for exploring EL not only in bacterial sepsis-induced ALI but also in hyperinflammatory viral pneumonia [24].

Mechanistic studies further suggested that the anti-inflammatory effects of EL were associated, at least in part, with regulation of the PI3K–Akt and NF-κB signaling pathways. Western blot analysis showed that EL reduced the phosphorylation levels of PI3K, Akt, IκBα, and p65 in both in vivo and in vitro models. Given the important roles of the PI3K–Akt cascade in macrophage activation and the NF-κB pathway in transcriptional regulation of proinflammatory mediators [25,26], modulation of these pathways may contribute to the attenuation of inflammatory responses and cytokine amplification by EL.

Among the compounds identified in EL, hyperoside was prioritized for further investigation based on the molecular docking results. Notably, hyperoside was the only compound predicted to interact favorably with EGFR, PI3K, and Akt simultaneously within the EGFR–PI3K–Akt axis. Considering the potential relevance of this pathway to inflammatory responses and virus-associated lung injury, hyperoside was selected as a representative candidate compound of EL for subsequent validation. Molecular docking analysis suggested favorable interactions between hyperoside and EGFR, PI3K, and Akt, and molecular dynamics simulations further supported the stability of these complexes, providing a possible molecular basis for its observed pharmacological effects.

In addition to its pronounced anti-inflammatory activity, hyperoside also showed protective effects in the pseudovirus-induced lung injury model. Network pharmacology and molecular docking analyses predicted that hyperoside may interact with several COVID-19-related targets, including ACE2, Mpro, and RdRp [27]. Molecular dynamics simulations further supported stable interactions with ACE2 and Mpro, whereas RdRp was not evaluated because of the lack of a suitable structural model in the Protein Data Bank. Based on these computational predictions, we further assessed the in vivo relevance of hyperoside in a SARS-CoV-2 pseudovirus-induced lung injury model. The results showed that hyperoside alleviated pseudovirus-associated lung injury, reduced ACE2 protein expression, and downregulated EGFR, PI3K, and Akt mRNA levels in lung tissues. These findings suggest that the protective effects of hyperoside may be associated with modulation of host inflammatory responses and ACE2-/EGFR/PI3K/Akt-related signaling molecules involved in lung injury, rather than direct evidence of antiviral activity.

Taken together, this study showed that EL alleviated ALI through multi-component, multi-target, and multi-pathway actions, including suppression of inflammatory responses and regulation of key signaling pathways. These findings provide additional pharmacological support for the traditional use of EL and suggest its potential value for further investigation in sepsis-associated lung injury and virus-associated inflammatory lung injury.

Nevertheless, several limitations should be acknowledged. First, although hyperoside was identified as a representative candidate compound, the contributions of other EL constituents and their potential interactions remain unclear. Second, the current study relied mainly on sepsis-associated and inflammatory injury models, and the pseudovirus model does not fully recapitulate authentic viral infection; therefore, further validation in live-virus infection models is needed. Third, pharmacokinetic, ADME, and toxicological evaluations of EL and its active constituents were not performed in the present study. In addition, the EL extract used here was not standardized by quantitative determination of hyperoside or other marker compounds, and the administered doses represent crude extract-equivalent dosing intended primarily for pharmacological exploration. These issues should be addressed in future studies to better define the material basis, safety profile, and translational potential of EL.

## 5. Conclusions

*Eupatorium lindleyanum* DC. exerts protective effects against sepsis-associated acute lung injury through coordinated multi-component and multi-target actions. In both in vivo and in vitro models, EL alleviated pulmonary edema, inflammatory cell infiltration, tissue injury, and excessive cytokine production. Mechanistically, EL suppressed macrophage activation and regulated key inflammatory signaling pathways, particularly the PI3K–Akt and NF-κB pathways, which may contribute to attenuation of the inflammatory cascade associated with cytokine storm. Integrative molecular docking and molecular dynamics analyses identified hyperoside as a representative candidate bioactive constituent with favorable predicted interactions with EGFR, PI3K, and Akt, suggesting a possible molecular basis for the observed anti-inflammatory effects. Moreover, combined computational analyses and pseudovirus-based in vivo validation suggest that hyperoside may be associated with modulation of SARS-CoV-2-related targets and lung injury-related signaling molecules, indicating potential relevance in virus-associated inflammatory lung injury. Collectively, these findings support EL as a natural multi-target candidate for further investigation in SALI and virus-associated inflammatory lung injury. Nevertheless, further studies employing authentic viral infection models, together with comprehensive pharmacokinetic characterization and systematic safety evaluation, are needed to better define its translational potential.

## Data Availability

The original contributions presented in this study are included in the article/Supplementary Materials. Further inquiries can be directed to the corresponding authors.

## Supplementary Materials

The following supporting information can be downloaded at https://www.mdpi.com/article/10.3390/cimb48030333/s1.

## Abbreviations

The following abbreviations are used in this manuscript:

ALI	Acute lung injury
BALF	Bronchoalveolar lavage fluid
BCA	Bicinchoninic acid assay
CLP	cecal ligation and puncture
CO_2_	Carbon dioxide
CSS	Cytokine storm syndrome
DAMPs	Damage-associated molecular patterns
EL	*Eupatorium lindleyanum* DC.
ELISA	Enzyme-linked immunosorbent assay
IκBα	Inhibitor of nuclear factor kappa B alpha
KEGG	Kyoto Encyclopedia of Genes and Genomes
NF-κB	Nuclear factor kappa-B
PAMPs	Pathogen-associated molecular patterns
PCA	Principal component analysis
PPI	Protein–protein interaction
Rg	Radius of gyration
RMSD	Root mean square deviation
RMSF	Root mean square fluctuation
SALI	Sepsis-induced acute lung injury
SASA	Solvent-accessible surface area
