# Supplementary material for: Eupatorium lindleyanum DC. Suppresses Cytokine Storm by Inhibiting NF-κB and PI3K–Akt Signaling in Sepsis-Associated and Virus-Related Acute Lung Injury
Source: Curr Issues Mol Biol. 2026 Mar 21;48(3):333. doi: 10.3390/cimb48030333 (PMC13024981; doi:10.3390/cimb48030333)
Supplement: Supplementary file 1 [file cimb-48-00333-s001.zip › Reporting Guidelines Information.pdf]

This study was reported in accordance with the ARRIVE 2.0 guidelines and the MDAR framework to ensure transparency and reproducibility.

1. Study design: In vivo CLP-induced SALI rat model and in vitro LPS-stimulated RAW264.7 macrophages to evaluate the protective effects of *Eupatorium lindleyanum* DC. (EL). Groups included sham/control, CLP model, EL (dose-graded), and DEX (positive control). Experimental units: single animal (*in vivo*) and single well/dish (*in vitro*).

2. Sample size: Animal experiments used n = 6 per group, based on prior studies and power considerations.

3. Inclusion & exclusion criteria: Healthy male SD rats within the target weight range were included; animals with failed modeling or unrelated illness were excluded.

4. Randomisation: Animals were randomly assigned using a random-number table method.

5. Blinding/Masking: Histological and biochemical assessments were performed blinded to group allocation.

6. Outcome measures: Primary outcomes included lung injury indices (histopathology, lung coefficient, wet/dry ratio), cytokines (TNF-α, IL-6, IL-1β), and activation of NF-κB and PI3K-Akt pathways; secondary outcomes included network pharmacology/docking/MD support.

7. Statistical methods: Data are mean ± SD; one-way ANOVA with Tukey post hoc test; significance at $p < 0.05$.

8. Experimental animals: Male Sprague–Dawley rats (6–8 weeks, 180–220 g) from the Animal Center of Chongqing Medical University (License SYXK [Yu] 2022-0010).

9. Experimental procedures: SALI was induced by CLP and treated with EL at indicated doses; RAW264.7 cells were stimulated by LPS and treated with EL as specified in Methods.

10. Results: All group data, statistics, and representative figures are reported in full in the Results/Figures and Supplementary sections in line with ARRIVE.
