# Supplementary figures and images for: Eupatorium lindleyanum DC. Suppresses Cytokine Storm by Inhibiting NF-κB and PI3K–Akt Signaling in Sepsis-Associated and Virus-Related Acute Lung Injury
Source: Curr Issues Mol Biol. 2026 Mar 21;48(3):333. doi: 10.3390/cimb48030333 (PMC13024981; doi:10.3390/cimb48030333)

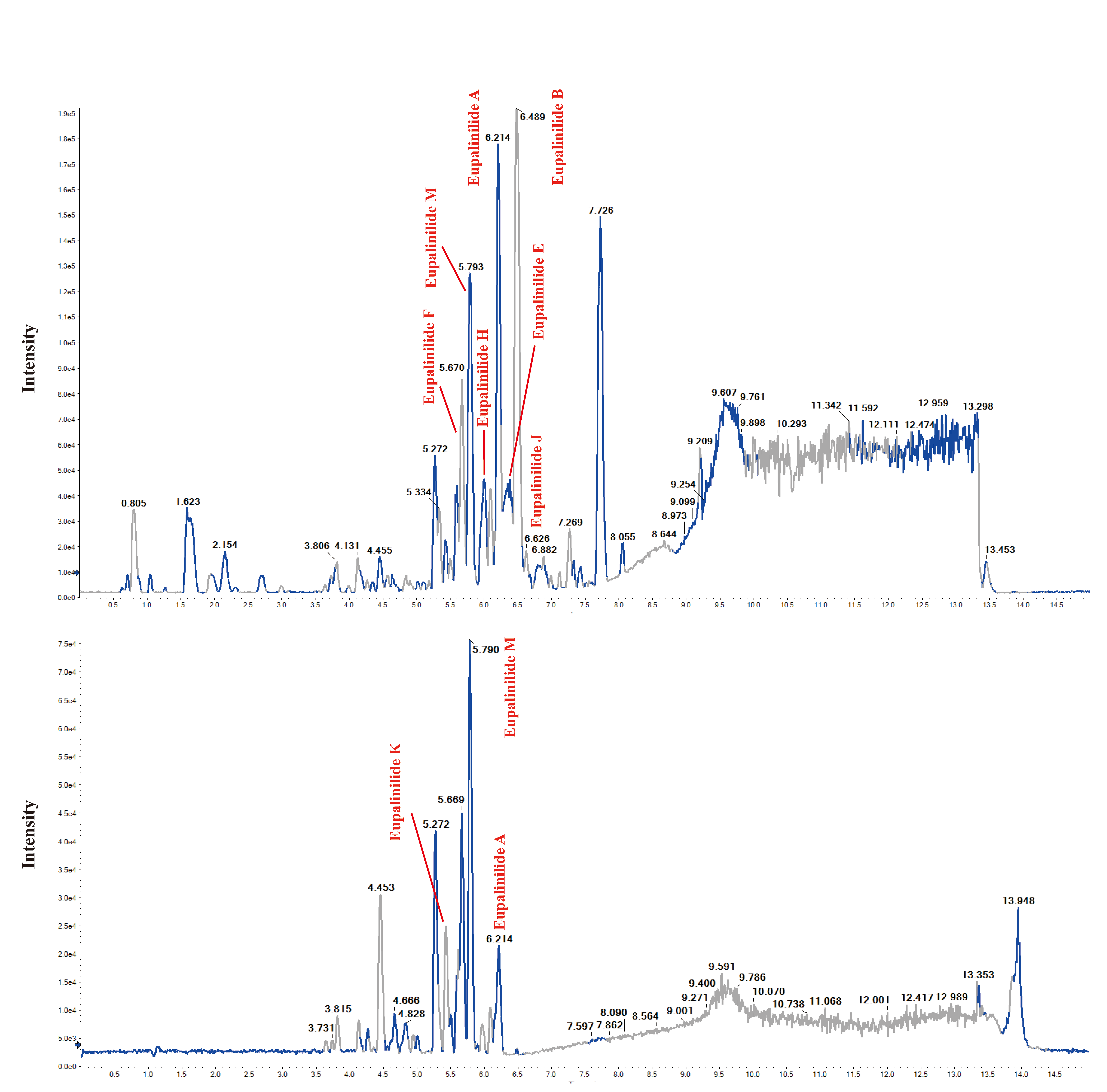

Supplement: Supplementary file 1 [file cimb-48-00333-s001.zip › Supplementary Figure S1. Chemical composition of EL.png]
